# Training programs in communication skills to improve self-efficacy for health personnel: Protocol for a systematic review and meta-analysis

**DOI:** 10.1097/MD.0000000000016697

**Published:** 2019-08-16

**Authors:** Ádala Nayana de Sousa Mata, Kesley Pablo Morais de Azevedo, Liliane Pereira Braga, Gidyenne Christine Bandeira Silva de Medeiros, Victor Hugo de Oliveira Segundo, Isaac Newton Machado Bezerra, Isac Davidson Santiago Fernandes Pimenta, Ismael Martínez Nicolás, Grasiela Piuvezam

**Affiliations:** a Multicampi School of Medical Sciences of Rio Grande do Norte, Federal University of Rio Grande do Norte, Caicó/RN; b Post-graduate Program in Public Health, Health Science Center, Federal University of Rio Grande do Norte, Natal/RN; c Department of Nutrition,; d Department of Public Health, Federal University of Rio Grande do Norte, Natal/RN, Brazil; e Department of Health Sciences, Catholic University San Antonio de Murcia, Murcia, Spain.

**Keywords:** communication, health personnel, self-efficacy, systematic review protocol, training

## Abstract

**Background:**

Patient-centered care should be the focus of health services, where improvements in the communication skills of health professionals promote excellent health and quality care. Thus, this study is a protocol for a systematic review and meta-analysis to evaluate the effectiveness of training programs in communication skills to promote self-efficacy in the communication of health personnel.

**Methods:**

This systematic review protocol is conducted using the Preferred Reporting Items for Systematic Reviews and Meta-Analyzes (PRISMA) statement guidelines and the Cochrane Handbook of Systematic Reviews of Interventions. The review should include studies carried out with health professionals who have undergone training in communication skills aimed at promoting their self-efficacy. Clinical trials (randomized, non-randomized), community trials, and quasi-experimental studies should be included. Therefore, the comprehensive search strategy will be conducted in the following databases: PubMed/Medline, Scopus, Web of Science, EMBASE, Science Direct, CINAHL, PsycINFO, and the Cochrane Central Register of Controlled Trials (CENTRAL). Two independent reviewers will conduct all study selection procedures, data extraction, and methodological evaluation, and disagreements will be referred to a third reviewer. RevMan 5.3 software will be used to gather data and perform the meta-analysis if possible.

**Results:**

This systematic review will provide evidence on more effective programs for communication skills training and will consider information such as duration, educational strategies, assessment measures, and outcomes that promote health worker self-efficacy.

**Discussion:**

This systematic review should provide evidence for effective communication skills training for health professionals in order to guide new strategies for quality care.

**Dissemination and ethics:**

The findings of this scoping review will be disseminated in print, at conferences, or via peer-reviewed journals. Ethical approval is not necessary as this paper does not involve patient data.

**Systematic review registration:**

PROSPERO CRD42019129384.

## 1 Introduction

The social and political changes that have occurred in this century point to the need for health systems that provide quality care and prioritize people in providing for their health and well-being.^[1]^ Thus, the literature indicates that high-quality health systems are structured on four principles: they are people-centered, equitable, resilient, and efficient.^[2]^

With regard specifically to person-centered care, the needs, preferences, and values of individuals should be considered in the transmission of information, involvement in decision-making, and respectful and responsible treatment.^[3]^ This care should be guided by dignity, compassion, and respect for people, as well as being a coordinated and personalized service, ensuring support to people in the recognition and development of their strengths and abilities.^[4]^

Health innovation processes that prioritize people's needs and values are increasingly being recognized in high- and low-income countries. These processes bring benefits in terms of health outcomes and the satisfaction of health professionals at all levels of the system,^[5]^ whether through the creation and encouragement of collective engagement, common values, good communication, teamwork, or transparency.^[6]^ The literature shows a consensus that communication between the professional and the patient is a key element in achieving patient-centered care.^[7]^

Given this context, the health sector is subject to strong pressure to change due to its organization, the lack of recognition of professional performance, and the deterioration of relationships with patients; therefore, the need for qualifications for its workers is emerging.^[8]^ It is believed that new strategies are necessary when approaching professionals because the complexity of health challenges requires innovative and creative methodologies for problem-solving as well as for the basis of available biomedical evidences.^[9]^

Thus, from the perspective of a collaborative work that places the user and their needs at the center of care, professionals need to broaden their practice to extend to one shared with professionals from other areas to have the potential to improve the quality of health care and to increase rationality related to health system costs.^[10]^ It is observed that through effective communication, professionals become familiar with the needs of their patients and can, therefore, offer health services of a higher quality.^[11]^ Thus, improving communication skills has been shown to be an important strategy for resource management, team training, and health care.^[12]^

The performance of professionals can be influenced by self-efficacy, that is, by how they perceive their abilities and evaluate their functions; what determines how they feel and think can be the strongest predictor of many behaviors, skills, and competences.^[13]^ Individuals with higher self-efficacy tend to view outcomes as positive because they believe in their own ability to communicate and perform their activities, thus making their performance more successful and satisfying.^[14]^

With this understanding, programs that address the issue of self-efficacy should be evaluated for clinical changes in attitudes and the development of confidence in communication competence itself as necessary conditions for improvement in health indicators.^[15,16]^ Understanding that communication skills are a central component of the health professional's routine and are decisive for the quality of the relationship with their patients,^[17]^ improvement programs for the development of these skills should be analyzed in order to identify better training strategies for professionals.

## 2 Objective

To describe the protocol of a systematic review and meta-analysis to identify which training programs in communication skills are effective in promoting self-efficacy in the communications of health personnel.

## 3 Methods and analysis

### 3.1 Study registration

This systematic review has been registered on PROSPERO (CRD42019129384), and will develop in accordance with the Preferred Reporting Items for Systematic Reviews and Meta-Analyses (PRISMA) statement guidelines^[18]^ and the Cochrane Handbook of Systematic Reviews of Interventions.^[19]^

### 3.2 Study selection criteria

#### 3.2.1 Types of studies

Clinical trials (randomized, non-randomized), community trials and quasi-experimental studies should be included.

#### 3.2.2 Types of participants

Health personnel from different health contexts should be included.

#### 3.2.3 Types of interventions

Typically, the training programs use different durations and strategies, such as reading texts, simulation, role play, etc. In this study, structured interventions to improve the communication skills of health professionals should be considered, with a definition of content, time, and evaluation of the results associated with improvement in professional performance (self-efficacy). The results of different communication skills training programs will be compared in this review.

#### 3.2.4 Types of outcomes

The results may include: 1. Improvement in self-efficacy in the communication skills of professionals; 2. Improvement in the communication skills of health professionals; 3. Improvement in the behavior or attitude of health professionals.

### 3.3 Search strategy

This systematic review will summarize evidence published by primary trials through a comprehensive search in the following databases: PubMed/Medline, Scopus, Web of Science, EMBASE, Science Direct, CINAHL, PsycINFO and the Cochrane Central Register of Controlled Trials (CENTRAL). The search strategy results from a combination of free text search terms and Medical Subject Headings (MeSH), text words, and keywords. The following key words will be used:

word group 1: health personnel OR health care providers OR health care workers

AND word group 2: communication OR empathy OR clinical competence OR clinical skills OR professional patient relations OR patient-centered care

AND word group 3: education OR training program OR workshop

AND word group 4: self-efficacy.

For example, the full search strategy for PubMed will be:

(”health personnel” OR ”health care providers” OR ”health care workers”) AND (”communicat∗” OR ”communication” OR ”empathy” OR ”clinical skills” OR ”professional patient relations” OR ”patient-centered care”) AND (”education” OR ”training program” OR ”workshop”) AND (”self-efficacy”).

The search terms used for the formation of the search equations will be combined with specific filters for each database. There will be no limitations of time and language in the searches performed.

### 3.4 Study selection

**Figure 1:**
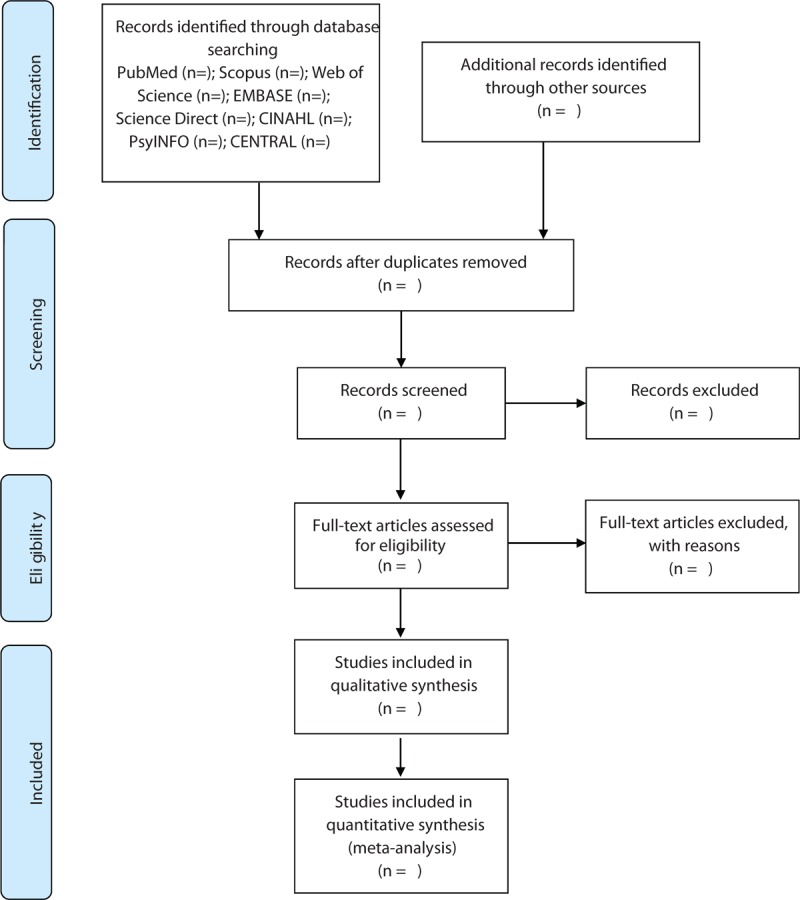
Flow diagram of study selection process.

Two reviewers will independently select the studies by scanning titles and summaries, and reading full texts if it is necessary according to the predefined eligibility criteria. Any disagreements regarding study selection will be solved by consulting a third reviewer. The whole process of study selection is summarized as a flowchart in Figure 1.

#### 3.4.1 Inclusion criteria

The studies must:

1. Be focused on communication skills training,
2. Be performed with health professionals,
3. Report a change in professional self-efficacy or other attitudinal and behavioral changes,
4. Be clinical trials (randomized, non-randomized), community trials, and quasi-experimental studies.

#### 3.4.2 Exclusion criteria

The studies must not:

1. Be conducted with undergraduate or graduate students,
2. Be interventions performed by mindfulness programs or using psychotherapy,
3. Lack full descriptions of the intervention or results.

### 3.5 Data extraction and management

Two authors will independently screen the search results using titles and abstracts, and full text. Duplicates and reviews will be removed from the database. Reviewers will then go through the full texts to determine whether they meet the inclusion criteria. References cited in articles will be further reviewed to locate any additional relevant articles not retrieved within the primary search. Discrepancies will be resolved by a third reviewer. The selection of the study is summarized in a PRISMA flow diagram.^[18]^

Data collected will include relevant information on population characteristics, study setting, type of communication education strategy used (brief description), study methods (length of intervention, length of follow-up, data collection points, inclusion criteria and method of randomization, if applicable), supporting evidence for educational strategy, effectiveness measurements, a description of each of the interventions and of each of the comparators, and the outcomes of significance to the review question.

### 3.6 Dealing with missing data

If the extractions are unclear or incomplete, we will attempt to obtain any missing data by contacting the first or corresponding authors or co-authors of an article via phone, email, or post. If we fail to receive any necessary information, the data will be excluded from our analysis and will be addressed in the discussion section.

### 3.7 Risk of bias assessment

Two authors will independently evaluate the risk of bias for each included article on the basis of the Cochrane Handbook for Systematic Reviews of Interventions^[19]^ to assess random sequence generation, allocation concealment, blinding of participants, and outcome assessment. In addition, incomplete outcome data, selective reporting, funding, and potential for conflicts of interest associated with the individual trials will also be considered. The risk of bias will be rated using predetermined criteria as follows: low, high, or unclear. The reviewers will be trained and calibrated beforehand to ensure uniformity in the evaluation of the criteria, and the Kappa index will be applied for agreement analysis.

### 3.8 Quality of evidence rating

The evaluation of the evidence and the strength of the recommendations of the studies will assessed with the Grading of Recommendations Assessment, Development, and Evaluation tool (GRADE).^[20]^

### 3.9 Data synthesis

The systematic review will describe the content of the included studies, such as population characteristics, context of the study, type of communication education strategy used (brief description), intervention data (duration, follow-up), evaluation measures, and significance of results.

All data from included studies will be analyzed by Review Manager (RevManV.5.3.3). Heterogeneity between trial results will be evaluated using a standard X^2^ test with a significance level 0.05. To assess heterogeneity, we plan to compute the I^2^ statistic, which is a quantitative measure of inconsistency across studies. A value of 0% indicates no observed heterogeneity, whereas I^2^ values of 50% indicate a substantial level of heterogeneity. If possible, funnel plots will be used to assess the presence of potential reporting biases. A linear regression approach will be used to evaluate funnel plot asymmetry.

## 4 Discussion

Patient-centered care involves techniques that seek to deepen and understand patients’ concerns, ideas, expectations, needs, and feelings, taking into consideration the psychosocial context in the search for a common understanding of the problem and treatment so that the patient is an active agent in the decision-making.^[21]^ It is imperative that the training of health professionals is aimed at ensuring qualified listening and the reception necessary for inclusion of the patient as an active agent in the care process.

In this sense, training in communication skills is a possible path and through this, different intervention strategies are used, such as songs, films, conversation wheels, video recording, direct observation, feedback, role-play, and simulations. These strategies are recognized as effective in developing skills for person-centered care,^[17,22,23,24]^ with positive results in both short- and long-term training.^[7]^

Studies conducted in recent years have revealed that adequate clinical communication has a positive influence on patients and can improve their satisfaction, health behaviors, and care costs.^[25,26,27]^ Jansen and Rosenbaum^[28]^ demonstrated that effective physician-patient communication improves patient health outcomes and adherence rates, and contributes to the recovery from health problems.

Studies addressing the impact of training on patient satisfaction did not show consensus. In this sense, the results of the study by Ehrstedt et al^[21]^ show that the centralization of care can positively affect patient satisfaction. However, other studies provide evidence that the effects of communication training for clinicians are small or inconclusive.^[29,30]^

Nevertheless, training interventions appear to be effective in improving the ability of practitioners to demonstrate empathy and discuss emotions.^[30]^ In addition, Burt's study^[31]^ presents the need for health professionals to reflect on their skill- and communication-related performance as part of their professional development, by considering the evaluations of both their work team and patients.

Interventions for health professionals are largely successful in transferring new patient-centered care skills^[7]^ and present positive correlations with those who exhibit increased levels of self-efficacy.^[32]^ However, further research is needed to directly assess the effects of interventions targeted only at providers.^[7]^ Thus, this systematic review intends to evaluate the results of training in communication skills conducted for different professional categories in order to identify effective strategies to improve the self-efficacy of professionals and to enable the formulation of new work strategies aimed at care centered on the patient and quality of care.

## Acknowledgment

We thank the Federal University of Rio Grande do Norte (UFRN) and the Coordination of Improvement of Higher Education Staff (CAPES) to encourage the development of the doctoral project.

## Author contributions

**Conceptualization:** Ádala Nayana de Sousa Mata, Grasiela Piuvezam.

**Data curation:** Gidyenne Christine Bandeira Silva de Medeiros, Victor Hugo de Oliveira Segundo, Isaac Newton Machado Bezerra, Isac Davidson Santiago Fernandes Pimenta, Ismael Martínez Nicolás, Kesley Pablo Morais de Azevedo.

**Formal analysis:** Grasiela Piuvezam.

**Funding acquisition:** Grasiela Piuvezam.

**Methodology:** Ádala Nayana de Sousa Mata, Kesley Pablo Morais de Azevedo, Liliane Pereira Braga, Gidyenne Christine Bandeira Silva de Medeiros, Victor Hugo de Oliveira Segundo, Isaac Newton Machado Bezerra, Isac Davidson Santiago Fernandes Pimenta, Ismael Martínez Nicolás, Grasiela Piuvezam.

**Project administration:** Ádala Nayana de Sousa Mata, Grasiela Piuvezam.

**Resources:** Ádala Nayana de Sousa Mata, Liliane Pereira Braga, Gidyenne Christine Bandeira Silva de Medeiros, Victor Hugo de Oliveira Segundo, Ismael Martínez Nicolás, Kesley Pablo Morais de Azevedo.

**Supervision:** Ádala Nayana de Sousa Mata, Ismael Martínez Nicolás, Grasiela Piuvezam.

**Writing – original draft:** Ádala Nayana de Sousa Mata, Grasiela Piuvezam.

**Writing – review & editing:** Ádala Nayana de Sousa Mata, Kesley Pablo Morais de Azevedo, Liliane Pereira Braga, Gidyenne Christine Bandeira Silva de Medeiros, Victor Hugo de Oliveira Segundo, Isaac Newton Machado Bezerra, Isac Davidson Santiago Fernandes Pimenta, Ismael Martínez Nicolás, Grasiela Piuvezam.

Ádala Nayana de Sousa Mata orcid: 0000-0003-3929-2082.
